# A hybrid *de novo* assembly of the sea pansy (*Renilla muelleri*) genome

**DOI:** 10.1093/gigascience/giz026

**Published:** 2019-04-03

**Authors:** Justin B Jiang, Andrea M Quattrini, Warren R Francis, Joseph F Ryan, Estefanía Rodríguez, Catherine S McFadden

**Affiliations:** 1 Department of Biology, Harvey Mudd College, 1250 N. Dartmouth Ave., Claremont, CA 91711, USA; 2 Department of Biology, University of Southern Denmark, Campusvej 55, Odense M 5230, Denmark; 3 Whitney Laboratory for Marine Bioscience, University of Florida, 9505 Ocean Shore Blvd., St. Augustine, FL 32080, USA; 4 Division of Invertebrate Zoology, American Museum of Natural History, Central Park West at 79th St., New York, NY 10024, USA

**Keywords:** octocoral, hybrid assembly, gene prediction, Augustus, PacBio, MaSuRCA

## Abstract

**Background:**

More than 3,000 species of octocorals (Cnidaria, Anthozoa) inhabit an expansive range of environments, from shallow tropical seas to the deep-ocean floor. They are important foundation species that create coral “forests,” which provide unique niches and 3-dimensional living space for other organisms. The octocoral genus *Renilla* inhabits sandy, continental shelves in the subtropical and tropical Atlantic and eastern Pacific Oceans. *Renilla* is especially interesting because it produces secondary metabolites for defense, exhibits bioluminescence, and produces a luciferase that is widely used in dual-reporter assays in molecular biology. Although several anthozoan genomes are currently available, the majority of these are hexacorals. Here, we present a *de novo* assembly of an azooxanthellate shallow-water octocoral, *Renilla muelleri*.

**Findings:**

We generated a hybrid *de novo* assembly using MaSuRCA v.3.2.6. The final assembly included 4,825 scaffolds and a haploid genome size of 172 megabases (Mb). A BUSCO assessment found 88% of metazoan orthologs present in the genome. An Augustus *ab initio* gene prediction found 23,660 genes, of which 66% (15,635) had detectable similarity to annotated genes from the starlet sea anemone, *Nematostella vectensis*, or to the Uniprot database. Although the *R. muelleri* genome may be smaller (172 Mb minimum size) than other publicly available coral genomes (256–448 Mb), the *R. muelleri* genome is similar to other coral genomes in terms of the number of complete metazoan BUSCOs and predicted gene models.

**Conclusions:**

The *R. muelleri* hybrid genome provides a novel resource for researchers to investigate the evolution of genes and gene families within Octocorallia and more widely across Anthozoa. It will be a key resource for future comparative genomics with other corals and for understanding the genomic basis of coral diversity.

## Data Description

### Organism description

Octocorallia is a subclass of Anthozoa (phylum: Cnidaria) that includes 3 orders: Alcyonacea, Helioporacea, and Pennatulacea [1]. The Pennatulacea, commonly known as sea pens, are a monophyletic group [1, 2] and are the most morphologically distinct group of octocorals [1, 3]. Sea pens differ from other octocorals by exhibiting the most integrated colonial behavior, with colonies arising from an axial polyp that develops into a peduncle—used to anchor the animal into soft sediments or onto hard surfaces—and a rachis that supports secondary polyps [1, 3, 4]. There are 14 valid families of Pennatulacea distinguished by the arrangement of the secondary polyps around the rachis [1, 4]. The monogeneric family Renillidae Lamarck, 1816 consists of 7 species [5], unique because of their foliate colony growth form [1, 4].

*Renilla* is found naturally on sandy, shallow sea floors along the Atlantic and Pacific coasts of North and South America [3, 4, 6]. The brilliant bioluminescence and endogenous fluorescence of these animals have led to them becoming important organisms in microscopy and molecular biology. Isolated originally from *Renilla reniformis*, the enzyme luciferase (Renilla-luciferin 2-mono-oxygenase) is used in dual luciferase reporter assays, which are commonly used to study gene regulation and expression, signaling pathways, and the structure of regulatory genes [7, 8]. The green fluorescent protein from *Renilla* has medical applications as well as general molecular biology and imagery uses [9]. In addition, the compounds produced by *Renilla* for chemical defense [10] may be important sources for discovery of marine natural products [11]. Thus, a genome of the octocoral *Renilla* is highly valuable to the scientific community, providing a novel resource that has a range of important uses—from molecular biology to comparative genomics.

Due to the known difficulties of resolving lengthy repeat regions with Illumina-only data [12, 13], we used a hybrid assembly approach [13, 14], combining long-read Pacific Biosciences (PacBio) data with short-read Illumina data. Studies have shown that a hybrid approach results in a more complete assembly with less genome fragmentation [15–17]. Our hybrid approach used low-coverage PacBio reads (15x coverage) along with high-coverage Illumina HiSeq reads (105x coverage) to assemble a draft genome of *Renilla muelleri* Schultze in Kölliker, 1872, a sea pen common to shallow waters of the Gulf of Mexico [6].

## Methods and Results

### Data collection

A live specimen of *R. muelleri* was obtained from Gulf Specimen Marine Lab (Panacea, FL, USA), which collects specimens off the panhandle of Florida in the Gulf of Mexico. Upon receipt of the specimen, it was flash frozen in liquid nitrogen. Genomic DNA was then extracted using a modified cetrimonium bromide (CTAB) protocol [18]. A total of 5.6 µg of DNA was sent to Novogene (Sacramento, CA, USA) for library preparation and sequencing. Then 350 base pair (bp) insert DNA libraries that were polymerase chain reaction free were prepared and multiplexed with other organisms on 2 lanes of an Illumina HiSeq 2500 (150 bp paired-end [PE] reads). In addition, Illumina MiSeq and PacBio sequencing were performed at the Weill Cornell Medicine Epigenomics Core Facility in New York. For the Illumina MiSeq run, the *Renilla* library was prepared with TruSeq LT and then multiplexed with eight other corals and sequenced (300 bp PE reads, MiSeq v3 Reagent kit). For PacBio sequencing, a DNA library was prepared from 5 μg of DNA using the SMRTbell template prep kit v.1.0. Sequencing was carried out on 10 single-molecule real-time sequencing (SMRT) cells on an RSII instrument using P6-C4 chemistry. PacBio SMRT Analysis 2.3 subread filtering module was used to produce the subread files for assembly.

As part of another study, we sequenced total RNA from a congeneric species, *R. reniformis*. The specimen was collected alive on the beach in North Flagler County, Florida, USA. RNA was extracted from the whole adult colony and sequenced on a NextSeq500 (150 bp PE reads) instrument. Library preparation and sequencing were performed at the University of Florida's Interdisciplinary Center for Biotechnology.

### DNA read processing

A total of 246,744,426 PE reads were obtained from the HiSeq and 6,725,072 PE reads were obtained from the MiSeq. In total, we generated 39,029,185,500 bases of Illumina data. Adapters were trimmed from all raw Illumina reads using Trimmomatic v.0.35 (ILLUMINACLIP:2:30:10 LEADING:5 TRAILING:5 SLIDINGWINDOW:4:20 MINLEN:3; Trimmomatic, RRID:SCR_011848) [19], resulting in 38.98 gigabases (Gb) of reads. These trimmed Illumina reads were then filtered with Kraken v.1.0 (Kraken, RRID:SCR_005484) [20] using the MiniKraken 8GB database [21] to screen for possible microbial contamination. The MiniKraken database includes complete bacterial, archaeal, and viral genomes from RefSeq. A total of 960 megabases (Mb) were removed from the read files, resulting in 36.23 Gb of 150 bp reads and 1.79 Gb of 300 bp reads (Supplemental File 1).

A total of 1,227,306 PacBio subreads were obtained and screened against the National Center for Biotechnology Information environmental nucleotide database (env_nt.00 to env_nt.23) [22] using BLASTn v.2.2.31 (–evalue 1e–10, –out_fmt 5, RRID:SCR_001598) [23] to identify reads with environmental contaminants (Supplemental Files 1–2). The subreads that did not contain contaminants were extracted using MEtaGenome ANalyzer v.6.4.16 (MEGAN, RRID:SCR_011942) [24, 25], resulting in 5.22 Gb in 1,195,521 reads.

### RNA sequencing read processing

We generated 119,604,588 PE reads of RNA sequencing (RNA-Seq) data. We used Trimmomatic v.0.36 (–phred33, ILLUMINACLIP:/usr/local/Trimmomatic-0.32/adapters/TruSeq3-PE.fa:2:30:12:1:true, MINLEN:36; Trimmomatic, RRID:SCR_011848) [19] to remove Illumina adapters. Trinity v.2.4.0 (–seqType fq –max_memory 250G –CPU 6 –left trim.R1.fq –right trim.R2.fq –full_cleanup; Trinity RRID:SCR_013048) [26] was used to assemble the transcriptome.

### Hybrid genome assembly

Two hybrid *de novo* assemblies were performed, one with the Maryland Super-Read Celera Assembler v.3.2.6 (MaSuRCA, RRID:SCR_010691) [27] and the other with SPAdes v.3.11.0 (SPAdes, RRID:SCR_000131; k-mer lengths 21 33 55 77) [28]. The Benchmarking Universal Single-Copy Orthologs v.3.0.2 (BUSCO, RRID:SCR_015008) [29] program with default settings (e-value 0.01) was used to screen the *Renilla* genome assemblies for 978 orthologs from the Metazoan data set as a method to evaluate the completeness of each assembly. BUSCO used BLAST v.2.2.31 [23] and HMMER v.3.1.b2 (HMMER, RRID:SCR_005305) [30] in its pipeline. The stats.sh program from BBMAP v.36.14 (bbmap) [31] was used to generate general assembly statistics for genomes produced by both programs (Table 1).

**Table 1:** General statistics and BUSCO-completeness of both initial hybrid assemblies and the final hybrid assembly

Statistic	MaSuRCA hybrid	SPAdes hybrid	Final MaSuRCA hybrid
Scaffold total	4,984	725,809	4,925
Contig total	5,263	725,809	5,196
Scaffold sequence total	172,512,580	231,255,108	172,160,214
Contig sequence total	172.472 Mb	231.255 Mb	172.091 Mb
Scaffold L/N50	635/70.423 kb	33,702/1.007 kb	633/70.522 kb
Contig L/N50	687/64.492 kb	33,702/1.007 kb	684/64.781 kb
Maximum scaffold/contig length	513.145 kb	323.009 kb	513.151 kb
No. of scaffolds >50 Kb	960	14	961
% main genome in scaffolds >50 Kb	61.07%	0.95%	61.23%
Guanine-cytosine content	36.18%	36.97%	36.17%
%N bases	0.042%	0	0.040%
BUSCO assessment			
Complete	858 (87.73%)	508 (51.94%)	857 (87.63%)
Complete and single-copy	826 (84.46%)	493 (50.41%)	826 (84.46%)
Complete and duplicated	32 (3.27%)	15 (1.53%)	31 (3.17%)
Fragmented	36 (3.68%)	200 (20.45%)	36 (3.68%)
Missing	84 (8.59%)	270 (27.61%)	85 (8.69%)

Unmerged haplotypes were removed in the final assembly, which was also error corrected with Pilon.

The MaSuRCA assembly resulted in a 147-fold decrease in the number of scaffolds generated, and a 70-fold increase in the N50 contig size (70.423 kb) as compared to the SPAades assembly (1.007 kb); it also had more complete BUSCOs present (Table 1). Other statistics also indicate that the MaSuRCA assembly is much less fragmented than the SPAdes assembly (Table 1). Therefore, we used the MaSuRCA assembly in further analyses.

To improve the quality of the draft MaSuRCA assembly, six iterations of Pilon v.1.21 (Pilon, RRID:SCR_014731) [32] were used to fix assembly errors and fill assembly gaps. Bowtie2 v.2.3.2 (Bowtie2, RRID:SCR_016368) [33] was used to align Illumina HiSeq and Illumina MiSeq genomic reads to the draft assembly, and the resulting alignments were input to Pilon, which was run on default settings. A total of 52,668 SNPs were corrected, along with 14,702 small insertions and 11,841 small deletions (Supplemental Table S1).

To remove haploid contigs that were not merged during assembly, we ran BLASTn against the contigs themselves (–max_target_seqs 10, –evalue 1e–40) to find contigs that were highly similar. The custom script haplotypeblastn.py version 1.0 [34] filtered the BLASTn results by flagging matches that were >75% identical and >500 bp in length. The contigs that were identified as unmerged were subsequently removed using the select_contigs.pl script [35]. A total of 59 scaffolds, which amounted to 67 contigs and 384 kb, were removed from the assembly.

The bbmap program stats.sh was used to generate assembly statistics on the haplotype-removed assembly (i.e., “final assembly”; Table 1). BUSCO analysis using the metazoan orthologs was again used to estimate the completeness of the final assembly, with default settings and the flag –long, to produce higher quality training data for the downstream annotation. There were 857 (87.63%) orthologs present in the final assembly (Table 1). This final *R. muelleri* assembly was masked, using RepeatMasker v.open-4.0.6 (–species eukaryota –gccalc –div 50; RepeatMasker, RRID:SCR_012954) [36] for downstream gene annotation. The final annotation consists of 172,512,580 bp in 4,925 scaffolds.

### Genome annotation

Stampy v.1.0.31 (Stampy, RRID:SCR_005504) [37] was used to align 18.06 Gb of RNA-Seq data from *R. reniformis* to the masked genome to generate intron hints, which provide evidence for introns based on spliced alignments. The resulting bam file was processed by filtering out raw alignments using filterBam [38] per the recommended Augustus procedures [39]. A total of 1,837,637 intron hints were generated.

Augustus v.3.3 (–UTR = off –allow_hinted_splicesites = atac –alternatives-from-evidence = true; Augustus, RRID:SCR_008417) [40] was used to predict a gene model for *R. muelleri*. Augustus training was performed with the hint data from *R. reniformis* because it has been shown to improve *ab initio* predictions [40, 41]. The BUSCO-generated training data were also included to help predict a gene model. A modified extrinsic weight file was used in Augustus to penalize predicted introns that were unsupported by hint evidence and reward predicted introns that were supported by hint evidence by 1e2.

Augustus predicted 23,660 genes that had a mean exon length of 249 bp and a mean intron length of 524 bp as calculated by gfstats.py [42] (Table 2). BUSCO with the metazoan lineage (*–*m prot) orthologs was used to assess the quality of the prediction, finding 84.87% (830/978) orthologs (Table 2).

**Table 2:** Statistics for the gene model predicted by Augustus

Statistic	Value
Genes	23,660
Exons	140,384
Introns	117,838
Mean exon length	249
Exons per gene	5.93
Mean intron length	524
Introns per gene	4.98
BUSCO assessment	
Complete	830 (84.87%)
Complete and single-copy	798 (81.60%)
Complete and duplicated	32 (3.27%)
Fragmented	64 (6.54%)
Missing	84 (8.59%)

### Functional annotations

BLASTp v.2.2.31+ (–evalue 1e–10 –seg yes –soft_masking true –lcase_masking, BLASTp, RRID:SCR_001010) [23] was used to map the predicted gene models of *R. muelleri* to filtered protein models of another anthozoan, the sea anemone, *Nematostella vectensis* (Joint Genome Institute, v.1.0), which is used as a model organism [43]. A total of 63% (14,931) of the predicted genes (23,660) mapped to *N. vectensis* proteins (27,273). A custom python script, filterGenes.py [44], was used to filter the matches by selecting the highest bit score; in cases where bit scores were identical, the match with the highest percent length of all matches was used as a tiebreaker. Of the 14,931 genes that mapped to *N. vectensis* proteins, 12,279 genes were annotated with GO function, KOG function, and/or InterPro domains; 8,101 genes were assigned GO terms; 11,067 genes were assigned KOG functions; and 10,126 genes were assigned InterPro domains (Supplemental File 3). The 8,729 genes that did not hit *N. vectensis* proteins were remapped with BLASTp using a lower e-value (1e–5) and filtered with the aforementioned python script with the same settings; an additional 2,002 of the genes mapped to *N. vectensis*. Of these, 1,512 genes were annotated with GO functions, KOG functions, and/or InterPro domains (Supplemental File 3). The remaining 6,727 genes that did not match *N. vectensis* annotations were mapped to the UniProt database (UniProt, RRID:SCR_002380) [45, 46] with BLASTp (–evalue 1e–5), and 1,844 of these were assigned a UniProt function. In total, 79.36% (18,777/23,660) of the predicted gene models were mapped to either *N. vectensis* predicted proteins or the UniProt database, and 66.08% (15,635/23,660) of the predicted *Renilla* genes have either functional annotations or InterPro domain information associated with them.

We also used BLASTp (–evalue 1e–10 –seg yes –soft_masking true –lcase_masking) to map the predicted genes against a newer *N. vectensis* data set that was generated using RNA-Seq (hereafter called the Vienna data set) [47, 48]. A total of 63.40% (15,001) of the predicted genes (23,660) mapped to the Vienna data set (25,729) (Supplemental File 4). As above, the predicted genes that did not map were remapped with a lower e-value (1e–5), resulting in 2,071 additional predicted genes mapping to the *N. vectensis* Vienna data set. In total, 72.15% (17,072) of predicted genes mapped to the Vienna dataset. This data set did not have associated functional annotations. Combining all gene model annotation methods, 79.82% (18,886) of genes from the Augustus gene model were mapped to the Joint Genome Institute *N. vectensis* annotations, the *N. vectensis* Vienna data set, or the UniProt database (Supplemental Files 3–5).

### Genome assembly comparisons

**Figure 1:**
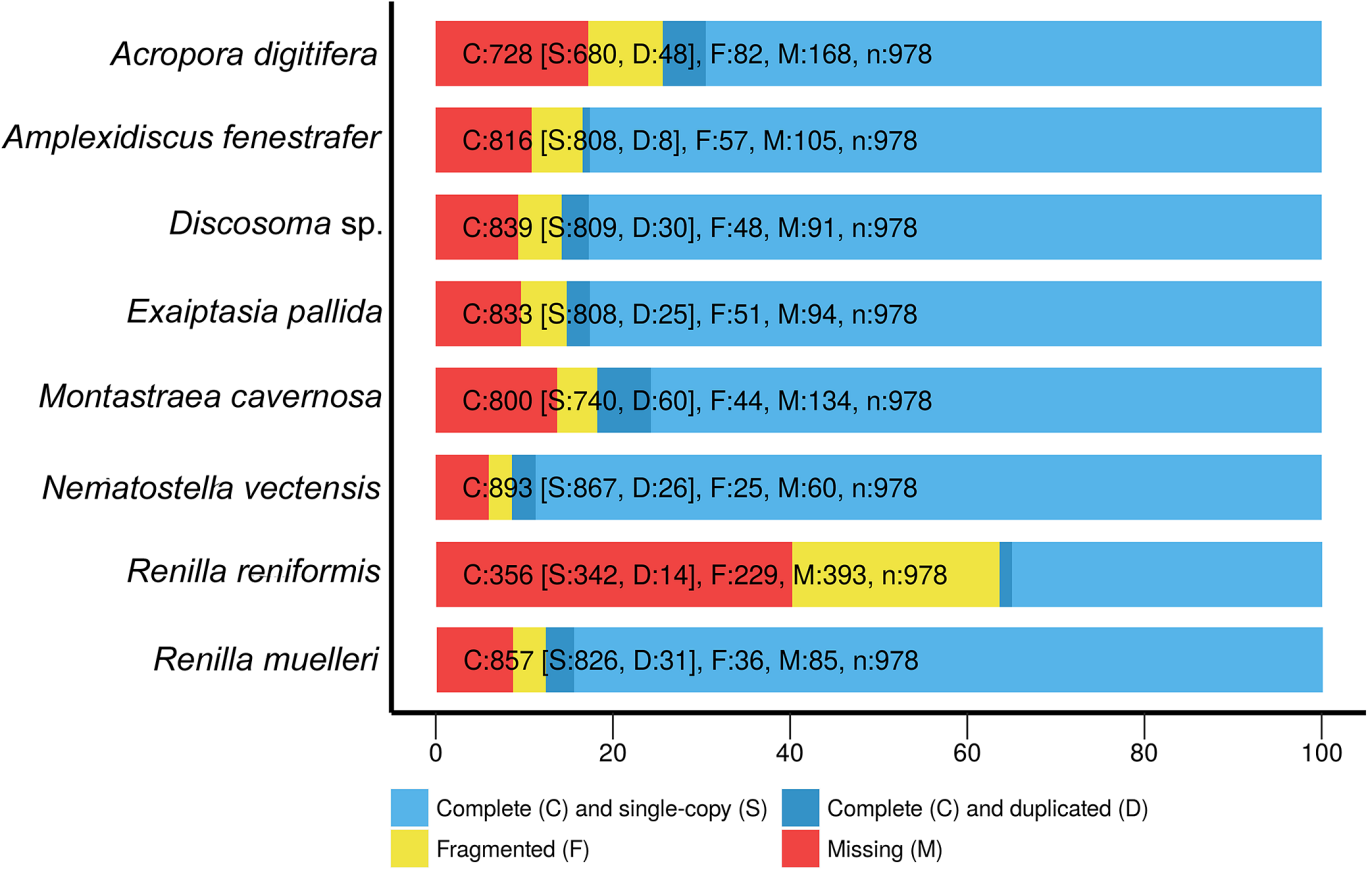
BUSCO-generated chart showing relative completeness of six hexacoral genomes, one octocoral genome, and the *Renilla muelleri* assembly.

We compared the *R. muelleri* genome assembly to previously published anthozoan (e.g., corals, anemones) genomes using a variety of assessment statistics (Supplemental Table S2). BUSCO was used with default parameters to assess the completeness of a draft *R. reniformis* genome and 6 hexacoral genomes (all masked with RepeatMasker with settings above) and compare these results to the hybrid *R. muelleri* assembly (Fig. 1). We found the BUSCO-completeness of our *R. muelleri* assembly (857 complete BUSCOs) to be most similar to the assembly of *N. vectensis* (893 complete BUSCOs) [49, 50]. BUSCOs from the other 5 hexacoral genomes were less complete, with complete BUSCOs ranging from 728 (*Acropora digitifera*) to 839 (*Discosoma* sp.) [50–58]. Only 800 complete BUSCOs were recovered from the other hybrid assembly, the hexacoral *Montastraea cavernosa* [58] The only other publicly available octocoral genome, *R. reniformis*, had considerably fewer complete BUSCOs (356, Fig. 1) [59]

The number of predicted genes was highly similar across all anthozoan genomes (Supplemental Table S2). The range of predicted genes was 21,372–30,360 across the six hexacorals. The number of predicted genes (23,360) for *R. muelleri* was similar to the 23,668 genes predicted for *A. digitifera*.

Interestingly, the genome size of *R. muelleri* is considerably smaller (172 Mb) than other coral genomes (256–448 Mb), although these genome sizes are minimum estimates due to the high number of scaffolds and fragmentary nature of the assemblies. Of the hexacorals, the anemone *Exaiptasia pallida* has the smallest genome size of 256 Mb, while the others have genome sizes >300 Mb. As indicated by Baumgarten et al. [57], *E. pallida* has smaller and less frequent introns. Similar to *E. pallida*, exon sizes were larger in *R. muelleri* (249 bp) compared with the hexacorals (208–230 bp). These results suggest that there may be comparatively fewer noncoding regions in *R. muelleri* because the number of predicted gene models in *R. muelleri* is similar to hexacorals, yet the exon sizes are larger and the genome size is smaller in *R. muelleri*. In addition, repetitive elements in the *R. muelleri* genome may be less frequent; however, this remains to be further examined. Alternatively, the comparatively small size of the *Renilla* genomes could be just because they are fragmented; more data might increase the size estimates.

We also compared the mitochondrial genome to the previously published mitogenome of *R. muelleri* [60]. We used BLASTn to search for the mitogenome among the contigs (included as the last contig in the assembly) and recovered the entire 18,641 bp circularized mitogenome. Compared to the published mitogenome, there were just two, single-bp differences and a 1-bp indel.

## Conclusions

We present an octocoral genome assembly and showcase the feasibility of the MaSuRCA hybrid assembler for marine invertebrate genomics. The *R. muelleri* genome may be one of the smallest anthozoan genomes discovered to date, yet it is comparable to other coral and anemone genomes in terms of predicted gene models. The identification of 88% of complete metazoan BUSCOs in the *R. muelleri* genome highlights that a high-quality genome assembly can be obtained from relatively low-coverage sequencing of short- and long-read data. Although more data are needed to further increase size and reduce number of scaffolds, and further functional annotation is needed, the genome of the sea pansy, *R. muelleri*, provides a novel resource for the scientific community to further investigations of gene family evolution, comparative genomics, and the genomic basis of coral diversity.

## Availability of supporting data and materials

The final hybrid assembly and predicted proteins generated by this study are in the *GigaDB* repository [61] and on the reefgenomics website [62]. Raw Illumina and PacBio reads are available in the National Center for Biotechnology Information's Sequence Read Archive (PRJNA491947). RNA-Seq reads have been uploaded to the European Nucleotide Archive (PRJEB28688).

## Additional files

**Supplemental Table S1**. Summary of Pilon changes per iteration.

**Supplemental Table S2**. *Renilla muelleri* genome assembly and annotation comparisons to other anthozoan genomes.

**Supplemental File 1**. List of reads that were regarded as potential microbial contaminants and removed from Illumina and PacBio data.

**Supplemental File 2**. Blast output of PacBio reads to env_nt database.

**Supplemental File 3**. Gene model annotations of *Renilla muelleri* using the *Nematostella vectensis* Joint Genome Institute filtered protein model.

**Supplemental File 4**. Gene annotations of *Renilla muelleri* using the *Nematostella vectensis* Vienna dataset.

**Supplemental File 5**. Reference file that includes annotations for the predicted gene models. This dataset includes GO terms, KOG IDs, and InterPro domains as annotated in the *Nematostella vectensis* filtered protein models (Joint Genome Institute).

## Abbreviations

bp: base pair; BUSCO: Benchmarking Universal Single-Copy Orthologs; Gb: gigabases; CTAB: cetrimonium bromide; MaSuRCA: Maryland Super-Read Celera Assembler; Mb: megabases; PE: paired end; Pacbio: Pacific Biosciences; RNA-Seq: RNA sequencing; SMRT: single-molecule real-time sequencing.

## Competing interests

The authors declare no competing interests.

## Funding

This study was funded by NSF-DEB Award 1457817 to C.S. McFadden and NSF-DEB Award 1457581 to E. Rodríguez. Additional funding came from startup funds from the University of Florida DSP Research Strategic Initiatives #00114464 and University of Florida Office of the Provost Programs to J.F. Ryan.

## Authors’ Contributions

J.J.: Conceptualization, investigation, formal analysis, software programming, methodology, validation, data curation, writing—original draft preparation, writing—review & editing, visualization.

A.M.Q.: Conceptualization, supervision, investigation, formal analysis, methodology, validation, data curation, writing—original draft preparation, writing—review & editing, visualization.

W.R.F.: Software programming, methodology, validation, writing—review & editing.

J.F.R.: Methodology, validation, data curation, writing—review & editing.

E.R.: Conceptualization, writing—review & editing.

C.S.M.: Conceptualization, formal analysis, supervision, writing—original draft preparation, writing—review & editing.

## Supplementary Material

GIGA-D-18-00366_Original-Submission.pdf

GIGA-D-18-00366_Revision-1.pdf

Response-to-Reviewer-Comments_Original-Submission.pdf

Reviewer-1-Report-Original-Submission -- Robert Steele, Ph.D. 12/5/2018 Reviewed

Reviewer-2-Report-Original-Submission -- Melody Clark 12/13/2018 Reviewed

Supplement_Files.zip
