# Supplementary material for: A hybrid de novo assembly of the sea pansy (Renilla muelleri) genome
Source: Gigascience. 2019 Apr 3;8(4):giz026. doi: 10.1093/gigascience/giz026 (PMC6446218; doi:10.1093/gigascience/giz026)
Supplement: GIGA-D-18-00366_Original-Submission.pdf [file giz026_giga-d-18-00366_original-submission.pdf]

GigaScience

A Hybrid de novo Assembly of the Sea Pansy (Renilla muelleri) Genome

--Manuscript Draft--

**Manuscript Number:**	GIGA-D-18-00366
**Full Title:**	A Hybrid de novo Assembly of the Sea Pansy (Renilla muelleri) Genome
**Article Type:**	Data Note
**Funding Information:**	National Science Foundation (1457817) — Dr. Catherine S. McFadden<br>National Science Foundation (1457581) — Dr. Estefanía Rodríguez<br>University of Florida DSP Research Strategic Initiatives (00114464) — Dr. Joseph F. Ryan<br>University of Florida Office of the Provost Programs (na) — Dr. Joseph F. Ryan

Abstract:

Background

Over 3,000 species of octocorals (Cnidaria, Anthozoa) inhabit an expansive range of environments, from shallow tropical seas to the deep-ocean floor. They are important foundation species that create coral “forests” which provide unique niches and three-dimensional living space for other organisms. The octocoral genus Renilla inhabits sandy, continental shelves in the subtropical and tropical Atlantic and eastern Pacific Oceans. Renilla is especially interesting because it produces secondary metabolites for defense, exhibits bioluminescence, and produces a luciferase that is widely used in dual-reporter assays in molecular biology. Although several cnidarian genomes are currently available, the majority are from hexacorals. Here, we present a de novo assembly of the R. muelleri genome, making this the first complete draft genome from an octocoral.

Findings

We generated a hybrid de novo assembly using the Maryland Super-Read Celera Assembler v.3.2.6 (MaSuRCA). The final assembly included 4,825 scaffolds and a haploid genome size of 172 Mb. A BUSCO assessment found 88% of metazoan orthologs present in the genome. An Augustus ab initio gene prediction found 23,660 genes, of which 66% (15,635) had detectable similarity to annotated genes from the starlet sea anemone, Nematostella vectensis, or to the Uniprot database. Although the R. muelleri genome is smaller (172 Mb) than other publicly available, hexacoral genomes (256-448 Mb), the R. muelleri genome is similar to the hexacoral genomes in terms of the number of complete metazoan BUSCOs and predicted gene models.

Conclusions

The R. muelleri hybrid genome provides a novel resource for researchers to investigate the evolution of genes and gene families within Octocorallia and more widely across Anthozoa. It will be a key resource for future comparative genomics with other corals and for understanding the genomic basis of coral diversity.

**Corresponding Author:**	Andrea M. Quattrini<br>Harvey Mudd College<br>Claremont, California UNITED STATES
**Corresponding Author Secondary Information:**	
**Corresponding Author's Institution:**	Harvey Mudd College
**Corresponding Author's Secondary Institution:**	
**First Author:**	Justin Jiang

Powered by Editorial Manager® and ProduXion Manager® from Aries Systems Corporation

First Author Secondary Information:	
Order of Authors:	Justin Jiang
	Andrea M. Quattrini
	Warren R. Francis
	Joseph F. Ryan
	Estefanía Rodríguez
	Catherine S. McFadden
Order of Authors Secondary Information:	
Additional Information:	
**Question**	**Response**
Are you submitting this manuscript to a special series or article collection?	No
**Experimental design and statistics**<br><br>Full details of the experimental design and statistical methods used should be given in the Methods section, as detailed in our Minimum Standards Reporting Checklist. Information essential to interpreting the data presented should be made available in the figure legends.<br><br>Have you included all the information requested in your manuscript?	Yes
**Resources**<br><br>A description of all resources used, including antibodies, cell lines, animals and software tools, with enough information to allow them to be uniquely identified, should be included in the Methods section. Authors are strongly encouraged to cite Research Resource Identifiers (RRIDs) for antibodies, model organisms and tools, where possible.<br><br>Have you included the information requested as detailed in our Minimum Standards Reporting Checklist?	Yes
**Availability of data and materials**	Yes

All datasets and code on which the conclusions of the paper rely must be either included in your submission or deposited in publicly available repositories (where available and ethically appropriate), referencing such data using a unique identifier in the references and in the "Availability of Data and Materials" section of your manuscript.<br><br>Have you have met the above requirement as detailed in our Minimum Standards Reporting Checklist?	

Powered by Editorial Manager® and ProduXion Manager® from Aries Systems Corporation

Manuscript
Click here to download Manuscript Jiang_GigaScience_final.docx

A Hybrid *de novo* Assembly of the Sea Pansy (*Renilla muelleri*) Genome

Justin Jiang[1], Andrea M. Quattrini[1*], Warren R. Francis[2], Joseph F. Ryan[3], Estefanía Rodríguez[4], Catherine S. McFadden[1]

[1]Department of Biology, Harvey Mudd College, 1250 N. Dartmouth Ave, Claremont, CA 91711, USA

[2] University of Southern Denmark, Dept. of Biology, Campusvej 55, Odense M 5230, Denmark

[3] Whitney Laboratory for Marine Bioscience, University of Florida, 9505 Ocean Shore Blvd. St. Augustine, FL 32080, USA

[4] Division of Invertebrate Zoology, American Museum of Natural History, Central Park West at 79th Street, New York, NY 10024, USA

Justin Jiang: jjiang990@gmail.com

Andrea Quattrini: aquattrini@g.hmc.edu

Warren R. Francis: wfrancis@biology.sdu.dk

Joseph F. Ryan: joseph.ryan@whitney.ufl.edu

Estefanía Rodríguez: erodriguez@amnh.org

Catherine S. McFadden: mcfadden@g.hmc.edu

*Corresponding Author

Abstract

Background: Over 3,000 species of octocorals (Cnidaria, Anthozoa) inhabit an expansive range of environments, from shallow tropical seas to the deep-ocean floor. They are important foundation species that create coral "forests" which provide unique niches and three-dimensional living space for other organisms. The octocoral genus *Renilla* inhabits sandy, continental shelves in the subtropical and tropical Atlantic and eastern Pacific Oceans. *Renilla* is especially interesting because it produces secondary metabolites for defense, exhibits bioluminescence, and produces a luciferase that is widely used in dual-reporter assays in molecular biology. Although several cnidarian genomes are currently available, the majority are from hexacorals. Here, we present a *de novo* assembly of the *R. muelleri* genome, making this the first complete draft genome from an octocoral.

Findings: We generated a hybrid *de novo* assembly using the Maryland Super-Read Celera Assembler v.3.2.6 (MaSuRCA). The final assembly included 4,825 scaffolds and a haploid genome size of 172 Mb. A BUSCO assessment found 88% of metazoan orthologs present in the genome. An Augustus *ab initio* gene prediction found 23,660 genes, of which 66% (15,635) had detectable similarity to annotated genes from the starlet sea anemone, *Nematostella vectensis*, or to the Uniprot database. Although the *R. muelleri* genome is smaller (172 Mb) than other publicly available, hexacoral genomes (256-448 Mb), the *R. muelleri* genome is similar to the hexacoral genomes in terms of the number of complete metazoan BUSCOs and predicted gene models.

Conclusions: The *R. muelleri* hybrid genome provides a novel resource for researchers to investigate the evolution of genes and gene families within Octocorallia and more widely across

Anthozoa. It will be a key resource for future comparative genomics with other corals and for understanding the genomic basis of coral diversity.

Keywords: octocoral, hybrid assembly, gene prediction, Augustus, PacBio, MaSuRCA

Data Description

Organism Description

Octocorallia is a subclass of Anthozoa (Phylum: Cnidaria) that is comprised of three orders: Alcyonacea, Helioporacea, and Pennatulacea [1]. The Pennatulacea, commonly known as sea pens, are a monophyletic group [1, 2] and are the most morphologically distinct group of octocorals [1, 3]. Sea pens differ from other octocorals by exhibiting the most integrated colonial behavior, with colonies arising from an axial polyp that develops into a peduncle—used to anchor the animal into soft-sediments or onto hard surfaces—and a rachis that supports secondary polyps [1, 3-4]. There are 14 valid families of Pennatulacea distinguished by the arrangement of the secondary polyps around the rachis [1, 4]. The monogeneric family Renillidae Lamarck, 1816 consists of seven species [5], unique because of their foliate colony growth form [1, 4].

Renilla is found naturally on sandy, shallow sea floors along the Atlantic and Pacific coasts of North and South America [3, 4, 6]. The brilliant bioluminescence and endogenous fluorescence of these animals have led to them becoming important organisms in microscopy and molecular biology. Isolated originally from *R. reniformis*, the enzyme luciferase (Renilla-luciferin 2-monooxygenase) is used in dual luciferase reporter assays, which are commonly used to study gene regulation and expression, signaling pathways, and the structure of regulatory

genes [7-8]. The green fluorescent protein from *Renilla* has medical applications as well as general molecular biology and imagery uses [9]. In addition, the compounds produced by *Renilla* for chemical defense [10] may be important sources for discovery of marine natural products [11]. Thus, a genome of the octocoral *Renilla* is highly valuable to the scientific community, providing a novel resource that has a range of important uses— from molecular biology to comparative genomics.

Due to the known difficulties of resolving lengthy repeat regions with Illumina-only data [12-13], we used a hybrid assembly approach [13-14], combining long-read Pacific Biosciences (PacBio) data with short-read Illumina data. Studies have shown that a hybrid approach results in a more complete assembly with less genome fragmentation [15-17]. Our hybrid approach used low coverage PacBio reads (15x coverage) along with high coverage Illumina HiSeq reads (105x coverage) to assemble a draft genome of *R. muelleri* Schultze in Kölliker, 1872, a sea pen common to shallow waters of the Gulf of Mexico [6].

Methods and Results

Data Collection

A live specimen of *R. muelleri* was obtained from Gulf Specimen Marine Lab (Panacea, FL, USA), which collects specimens off the panhandle of Florida in the Gulf of Mexico. Upon receiving the specimen, it was flash frozen in liquid nitrogen. Genomic DNA was then extracted using a modified CTAB protocol [18]. A total of 5.6 µg of DNA was sent to Novogene (Sacramento, CA, USA) for library preparation and sequencing. 350 bp insert DNA libraries that were PCR free were prepared and then multiplexed with other organisms on two lanes of an Illumina HiSeq 2500 (150 bp PE reads). In addition, Illumina MiSeq and PacBio sequencing

were performed at the Weill Cornell Medicine Epigenomics Core Facility in New York. For the Illumina MiSeq run, the *Renilla* library was prepared with TruSeq LT and then multiplexed with eight other corals and sequenced (300 bp PE reads, MiSeq v3 Reagent kit). For PacBio sequencing, a DNA library was prepared from 5 ug of DNA using the SMRTbell template prep kit v 1.0. Sequencing was carried out on 10 SMRT cells on a RSII instrument using P6-C4 chemistry. PacBio SMRT Analysis 2.3 subread filtering module was used to produce the subread files for assembly.

As part of another study, we sequenced total RNA from a congeneric species, *R. reniformis*. The specimen was collected alive on the beach in North Flagler County, Florida, USA. RNA was extracted from the whole adult colony and sequenced on a NextSeq500 (150 bp PE reads) instrument. Library preparation and sequencing were performed at the University of Florida's Interdisciplinary Center for Biotechnology.

DNA Read Processing

A total of 246,744,426 PE reads were obtained from the HiSeq and 6,725,072 PE reads were obtained from the MiSeq. In total, we generated 39,029,185,500 bases of Illumina data. Adapters were trimmed from all raw Illumina reads using Trimmomatic v.0.35 (*ILLUMINACLIP:2:30:10 LEADING:5 TRAILING:5 SLIDINGWINDOW:4:20 MINLEN:3*; Trimmomatic, RRID:SCR_011848) [19], resulting in 38.98 Gb of reads. These reads were then filtered with Kraken v.1.0 (Kraken, RRID:SCR_005484) [20] using the MiniKraken 8GB database [21] to screen for possible microbial, viral and archaeal contamination. A total of 960 Mb were removed from the read files, resulting in 36.23 Gb of 150 bp reads and 1.79 Gb of 300 bp reads.

A total of 1,227,306 PacBio subreads were obtained and screened against the NCBI environmental nucleotide database (env_nt.00 to env_nt.23) [22] using BLASTn v.2.2.31 (-*evalue 1e-10, -out_fmt 5*, RRID:SCR_001598) [23] to identify reads with environmental contaminants. The subreads that did not contain contaminants were extracted using MEtaGenome ANalyzer v.6.4.16 (MEGAN, RRID:SCR_011942) [24-25], resulting in 5.22 Gb in 1,195,521 reads.

RNA-Seq Read Processing

We generated 119,604,588 PE reads of RNA-Seq data. We used Trimmomatic (version 0.36 (-phred33, ILLUMINACLIP:/usr/local/Trimmomatic-0.32/adapters/TruSeq3-PE.fa:2:30:12:1:true, MINLEN:36; Trimmomatic, RRID:SCR_011848) [19] to remove Illumina adapters. Trinity v 2.4.0 (--seqType fq --max_memory 250G --CPU 6 --left trim.R1.fq --right trim.R2.fq --full_cleanup; Trinity RRID_SCR:013048) [26] was used to assemble the transcriptome.

Hybrid Genome Assembly

Two hybrid *de novo* assemblies were performed, one with the Maryland Super-Read Celera Assembler v.3.2.6 (MaSuRCA, RRID:SCR_010691) [27] and the other with SPAdes v.3.11.0 (SPAdes, RRID:SCR_000131; *k-mer lengths 21,33,55,77*) [28]. The Benchmarking Universal Single-Copy Orthologs v.3.0.2 (BUSCO, RRID:SCR_015008) [29] program with default settings was used to screen the *Renilla* genome assemblies for 978 orthologs from the Metazoan dataset as a method to evaluate the completeness of each assembly. BUSCO used BLAST v.2.2.31 [23] and HMMER v.3.1.b2 (HMMER, RRID:SCR_005305) [30] in its pipeline.

The stats.sh program from BBMAP v.36.14 (bbmap) [31] was used to generate general assembly statistics for genomes produced by both programs (Table 1).

The MaSuRCA assembly resulted in a 147-fold decrease in the number of scaffolds generated, and a 70-fold increase in the N50 contig size (70.522 KB) as compared to the SPades assembly (1.007 KB); it also had more complete BUSCOs present (Table 1). Other statistics also indicate that the MaSuRCA assembly is much less fragmented than the SPAdes assembly (Table 1). Therefore, we used the MaSuRCA assembly in further analyses.

To improve the quality of the draft MaSuRCA assembly, six iterations of Pilon v.1.21 (Pilon, RRID:SCR_014731) [32] were used to fix assembly errors and fill assembly gaps. Bowtie2 v.2.3.2 (Bowtie2, RRID:SCR_016368) [33] was used to align Illumina HiSeq and Illumina MiSeq genomic reads to the draft assembly, and the resulting alignments were input to Pilon, which was run on default settings. A total of 52,668 SNPs were corrected, along with 14,702 small insertions and 11,841 small deletions (Supplementary Table S1).

To remove haploid contigs that were not merged during assembly, we ran BLASTn against the contigs themselves *(-max_target_seqs 10, -evalue 1e-40*) to find contigs that were highly similar. The custom script *haplotypeblastn.py* version 1.0 [34] filtered the BLASTn results by flagging matches that were greater than 75% identical and longer than 500 bp in length. The contigs that were identified as unmerged were subsequently removed using the *select_contigs.pl* script [35]. A total of 59 scaffolds, which amounted to 67 contigs and 384 kb, were removed from the assembly.

The bbmap program stats.sh was used to generate assembly statistics on the haplotype-removed assembly [i.e., “final assembly”, (Table 1)]. BUSCO analysis using the metazoan orthologs was again used to estimate the completeness of the final assembly, with the flag *-long*

to produce higher quality training data for the downstream annotation. 857 (87.63%) orthologs were present in the final assembly (Table 1). This final *R. muelleri* assembly was masked, using RepeatMasker v.open-4.0.6 (*-species eukaryota -gccalc -div 50;* RepeatMasker, RRID:SCR_012954) [36], for downstream gene annotation. The final annotation consists of 172,512,580 bp in 4,925 scaffolds.

Genome Annotation

Stampy v.1.0.31 (Stampy, RRID:SCR_005504) [37] was used to align 18.06 Gb of RNA-Seq data from *R. reniformis* to the masked genome to generate intron hints. The resulting bam file was processed by filtering out raw alignments using *filterBam* [38] per the recommended Augustus procedures [39]. A total of 1,837,637 intron hints were generated.

Augustus v.3.3 (*--UTR=off --allow_hinted_splicesites=atac --alternatives-from-evidence=true;* Augustus, RRID:SCR_008417) [40] was used to predict a gene model for *R. muelleri*. Augustus training was performed with the hint data from *R. reniformis*, as it has been shown to improve *ab initio* predictions [40-41]. The BUSCO-generated training data was also included to help predict a gene model. A modified extrinsic weight file was used in Augustus to penalize predicted introns that were unsupported by hint evidence and reward predicted introns that were supported by hint evidence by 1e2.

Augustus predicted 23,660 genes that had an average exon length of 249 bp and an average intron length of 524 bp as calculated by *gfstats.py* [42] (Table 2). BUSCO with the metazoan lineage (*-m prot*) orthologs was used to assess the quality of the prediction, finding 84.87% (830/978) orthologs (Table 2).

Functional Annotations

BLASTp v.2.2.31+ (*-evalue 1e-10 -seg yes -soft_masking true -lcase_masking*, BLASTp, RRID:SCR_001010) [23] was used to map the predicted gene models of *R. muelleri* to filtered protein models of another anthozoan, the sea anemone, *Nematostella vectensis* (Joint Genome Institute, JGI, v 1.0) [43]. A total of 63% (14,931) of the predicted genes (23,660) mapped to *N. vectensis* proteins (27,273). A custom python script, *filterGenes.py* [44] was used to filter the matches by selecting the highest bit score; in cases where bit scores were identical, the match with the highest percent length of all matches was used as a tiebreaker. Of the 14,931 genes that mapped to *N. vectensis* proteins, 12,279 genes were annotated with GO function, KOG function and/or InterPro domains; 8,101 genes were assigned GO terms; 11,067 genes were assigned KOG functions; and 10,126 genes were assigned InterPro domains (Supplemental File 1). The 8,729 genes that did not hit *N. vectensis* proteins were remapped with BLASTp using a lower e-value (*1e-5*) and filtered with the aforementioned python script with the same settings; an additional 2,002 of the genes mapped to *N. vectensis*. Of these, 1,512 genes were annotated with GO functions, KOG functions and/or InterPro domains (Supplemental File 1). The remaining 6,727 genes that did not match *N. vectensis* annotations were mapped to the UniProt database (UniProt, RRID:SCR_002380) [45-46] with BLASTp (*-evalue 1e-5*), and 1,844 of these were assigned a UniProt function. In total, 79.36% (18,777/23,660) of the predicted gene models were mapped to either *N. vectensis* predicted proteins or the UniProt database, and 66.08% (15,635/23,660) of the predicted *Renilla* genes have either functional annotations or InterPro domain information associated with them.

We also used BLASTp (*-evalue 1e-10 -seg yes -soft_masking true -lcase_masking)* to map the predicted genes against a newer *N. vectensis* dataset that was generated using RNA-Seq

(hereafter called the Vienna dataset) [47-48]. A total of 63% (15,001) of the predicted genes (23,660) mapped to the Vienna dataset (25,729) (Supplemental File 2). As above, the predicted genes that did not map were remapped with a lower e-value (*1e-5),* resulting in 2,071 additional predicted genes mapping to the *N. vectensis* Vienna dataset. In total, 72.15% (17,072) of predicted genes mapped to the Vienna dataset. This dataset did not have associated functional annotations. Combining all gene model annotation methods, 79.82% (18,886) of genes from the Augustus gene model were mapped to the JGI *N. vectensis* annotations, the *N. vectensis* Vienna dataset, or the UniProt database (Supplemental Files 1-3).

Genome Assembly Comparisons

We compared the *R. muelleri* genome assembly to previously published anthozoan (e.g., corals, anemones) genomes using a variety of assessment statistics (Supplemental Table S2). BUSCO was used (*-m geno)* to assess the completeness of six hexacoral genomes and a draft *R. reniformis* genome and compare these results to the hybrid *R. muelleri* assembly (Fig. 1). We found the BUSCO-completeness of our *R. muelleri* assembly (857) to be more similar to the well-curated assembly of the model organism *N. vectensis* (893) [49-50] than to the other anthozoans. BUSCOs from the other five hexacoral genomes were less complete, with complete BUSCOs ranging from 728 (*Acropora digitifera*) to 839 (*Discosoma* sp.) [50-57]. Only 800 complete BUSCOs were recovered from the other hybrid assembly, the hexacoral *Montastraea cavernosa* [57]. The only other publicly-available octocoral genome, *R. reniformis*, had considerably fewer complete BUSCOs (356, Fig.1) [58].

The number of predicted genes was highly similar across all anthozoan genomes (Supplementary Table S2). The range of predicted genes was 21,372 to 30,360 across the six

hexacorals. The number of predicted genes (23,360) for *R. muelleri* was similar to the 23,668 genes predicted for *A. digitifera*.

Interestingly, the genome size of *R. muelleri* is considerably smaller (172 Mb) than other hexacoral genomes (256-448 Mb). Of the hexacorals, the anemone *Exaiptasia pallida* has the smallest genome size of 256 Mb, while the others have genome sizes >300 Mb. As indicated by [56], *E. pallida* has smaller and less frequent introns. Similar to *E. pallida*, exon sizes were larger in *R. muelleri* (249 bp) compared to the hexacorals (208 to 230 bp). These results suggest that there may be comparatively fewer non-coding regions in *R. muelleri* because the number of predicted gene models in *R. muelleri* is similar to hexacorals, yet the exon sizes are larger and the genome size is smaller in *R. muelleri.* In addition, repetitive elements in the *R. muelleri* genome may be less frequent, however, this remains to be further examined.

We also compared the mitochondrial genome to the previously published mitogenome of *R. muelleri* [59]. We used BLASTn to search for the mitogenome among the contigs (included as the last contig in the assembly) and recovered the entire 18,641 bp circularized, mitogenome. Compared to the published mitogenome, there were just two, single bp differences and one bp indel.

Conclusions

We present the first octocoral genome assembly and showcase the feasibility of the MaSuRCA hybrid assembler for marine invertebrate genomics. The *R. muelleri* genome is one of the smallest anthozoan genomes discovered to date, yet it is comparable to other coral and anemone genomes in terms of predicted gene models. The identification of 88% of complete metazoan BUSCOs in the *R. muelleri* genome highlights that a quality genome assembly can be

obtained from relatively low coverage sequencing of short and long read data. Although more data are needed to further increase size and reduce number of scaffolds, and further functional annotation is needed, the genome of the sea pansy, *R. muelleri*, provides a novel resource for the scientific community to further investigations of gene family evolution, comparative genomics, and the genomic basis of coral diversity.

Availability of supporting data

The final hybrid assembly and predicted proteins generated by this study are in the *Giga*DB repository [60] and on the reefgenomics website [61]. Raw Illumina and PacBio reads are available in NCBI's Sequence Read Archive (PRJNA491947). RNA-Seq reads have been uploaded to the European Nucleotide Archive (PRJEB28688).

Abbreviations

bp: base pair, BUSCO: Benchmarking Universal Single-Copy Orthologs, Gb: gigabp, Mb: megabp, MY: million years, PE: paired end, Pacbio: Pacific Biosciences

Additional Files

Supplementary Table S1. Summary of Pilon changes per iteration

Supplemental Table S2. *Renilla muelleri* genome assembly and annotation comparisons to other anthozoan genomes.

Supplemental File 1. Gene model annotations of *Renilla muelleri* using the *Nematostella vectensis* Joint Genome Institute filtered protein model.

Supplemental File 2. Gene annotations of *Renilla muelleri* using the *Nematostella vectensis*

Vienna dataset.

Supplemental File 3. Reference file that includes annotations for the predicted gene models. This dataset includes GO terms, KOG IDs, and InterPro domains as annotated in the *Nematostella vectensis* filtered protein models (Joint Genome Institute).

Competing interests

The authors declare no competing interests.

Funding

This study was funded by NSF-DEB Award 1457817 to C.S. McFadden and NSF-DEB Award 1457581 to E. Rodriguez. Additional funding came from startup funds from the University of Florida DSP Research Strategic Initiatives #00114464 and University of Florida Office of the Provost Programs to J.F. Ryan.

Authors' Contributions

Justin Jiang: Conceptualization, Investigation, Formal Analysis, Software Programming, Methodology, Validation, Data Curation, Writing - Original Draft Preparation, Writing - Review & Editing, Visualization

Andrea M. Quattrini: Conceptualization, Supervision, Investigation, Formal Analysis, Methodology, Validation, Data Curation, Writing - Original Draft Preparation, Writing - Review & Editing, Visualization

Warren R. Francis: Software Programming, Methodology, Validation, Writing - Review & Editing

Joseph F. Ryan: Methodology, Validation, Data Curation, Writing - Review & Editing

Estefania Rodriguez: Conceptualization, Writing - Review & Editing

Catherine S. McFadden: Conceptualization, Supervision, Writing - Original Draft Preparation, Writing - Review & Editing

Acknowledgements

We thank N. Alexander, C. Mason, and the Weill Cornell Medicine Epigenetics Core Facility and staff for MiSeq and PacBio sequencing. Thanks to M. Brugler, C. Schnitzler, and S. Herrera for advice. B. Macdonald generated the filterGenes.py script. We thank M. Heloski for collection of *Renilla reniformis* sample used for RNA-Seq.

References

1. Daly M, Brugler MR, Cartwright P et. al. The phylum Cnidaria: A review of phylogenetic patterns and diversity 300 years after Linnaeus. Zootaxa. 2007;1668:127-182.
2. McFadden CS, France SC, Sánchez JA et. al. A molecular phylogenetic analysis of the Octocorallia (Cnidaria: Anthozoa) based on mitochondrial protein-coding sequences. Molecular Phylogenetics and Evolution. 2006;41(3):513:527.
3. Williams GC. The global diversity of sea pens (Cnidaria: Octocorallia: Pennatulacea). PLoS One. 2011;6:e22747
4. Williams GC. Living genera of sea pens (Coelenterata: Octocorallia: Pennatulacea): illustrated key and synopsis. Zoological Journal of the Linnean Society. 1995;113:93-140.

5. World Register of Marine Species: World List of Octocorallia Renillidae. http://marinespecies.org/aphia.php?p=taxdetails&id=266953, Accessed 19 Aug 2018.
6. Cairns SD, Bayer FM. Octocorallia (Cnidaria) of the Gulf of Mexico. In: Felder DL, Camp DK, editors. Gulf of Mexico–Origins, Waters, and Biota. Volume 1. Biodiversity. College Station, Texas: Academic; 2009:321-331.
7. Sherf BA, Navarro SL, Hannah RR, Wood KV. Dual-luciferase reporter assay: an advanced co-reporter technology integrating firefly and Renilla luciferase assays. Promega Notes. 1996;56:2.
8. Saito K, Chang YF, Horikawa K et al. Luminescent Proteins for High-Speed Single-Cell and Whole-Body Imaging. Nature Communications. 2012; doi:10.1038/ncomms2248.
9. Stepanenko OV, Verkhusha VV, Kuznetsova IM, Uversky VN, Turoverov KK. Current Protein & Peptide Science. 2008; doi:10.2174/138920308785132668
10. Clavico EE, De Souza AT, Da Gama BA, Pereira RC. Antipredator defense and phenotypic plasticity of sclerites from *Renilla muelleri*, a tropical sea pansy. The Biological Bulletin, 2007;213(2):135-140.
11. Ledoux JB, Antunes A. Beyond the beaten path: improving natural products bioprospecting using an eco-evolutionary framework–the case of the octocorals. Critical Reviews in Biotechnology. 2018;38(2):184-198.
12. Pop M, Salzberg SL. Bioinformatics challenges of new sequencing technology. Trends in Genetics. 2008;24(3):142-149.
13. Koren S, Schatz MC, Walenz BP, Martin J, Howard JT, Ganapathy G, Phillippy AM. Hybrid error correction and de novo assembly of single-molecule sequencing reads. Nature biotechnology, 2012;30(7):693.

14. English AC, Richards S, Han Y et al. Mind the gap: Upgrading genomes with Pacific Biosciences RS long-read sequencing technology. PLoS ONE. 2012; doi:10.1371/journal.pone.0047768.

15. Bashir A, Klamer AA, Robins WP et al. A hybrid approach for the automated finishing of bacterial genomes. Nature Biotechnology. 2012; doi:10.1038/nbt.2288.

16. Giordano F, Aigrain L, Quail MA et al. De novo yeast genome assemblies from MinION, PacBio and MiSeq platforms. Scientific Reports. 2017; doi:10.1038/s41598-017-03996-z.

17. Tan MH, Austin CM, Hammer MP et al. Finding Nemo: hybrid assembly with Oxford Nanopore and Illumina reads greatly improves the clownfish (*Amphiprion ocellaris*) genome assembly. GigaScience. 2018; doi:10.1093/gigascience/gix137.

18. McFadden CS, Alderslade P, Ofwegen LP van, Johnsen H, Rusmevichientong A. Phylogenetic relationships within the tropical soft coral genera *Sarcophyton* and *Lobophytum* (Anthozoa, Octocorallia). Invertebrate Biology 2006;125:288-305.

19. Bolger AM, Lohse M, Usadel B. Trimmomatic: a flexible trimmer for Illumina sequence data. Bioinformatics 2014;30(15):2114–20.

20. Wood DE, Salzberg SL. Kraken: ultrafast metagenomic sequence classification using exact alignments. Genome Biology. 2014;15(3):R46.

21. Wood DE. Minikraken 8 GB database, Johns Hopkins University, https://ccb.jhu.edu/software/kraken/dl/minikraken_20171019_8Gb.tgz (August 7 2018, date last accessed)

22. National Center for Biotechnology Information: Trivial HTTP: env_nt.00 to env_nt.23. ftp://ftp.ncbi.nlm.nih.gov/blast/db/

23. Boratyn GM, Camacho C, Cooper PS et al. BLAST: a more efficient report with usability improvements. Nucleic Acids Research 2013;41(W1):W29–33.

24. Huson DH, Mitra S, Ruscheweyh HJ et al. Integrative analysis of environmental sequences using MEGAN4, Genome Research, 2011;21:1552-1560.

25. Huson DH, Auch AF, Qi J et al. MEGAN analysis of metagenomic data, Genome Research, 2007;17(3):377-86.

26. Haas BJ, Papanicolaou A, Yassour M, Grabherr M, Blood PD, Bowden J, MacManes MD. De novo transcript sequence reconstruction from RNA-seq using the Trinity platform for reference generation and analysis. Nature protocol. 2013;8(8):1494.

27. Zimin AV, Marçais G, Puiu D et al. The MaSuRCA genome assembler. Bioinformatics 2013;29(21):2669–77.

28. Bankevich A, Nurk S, Antipov D, et al. SPAdes: A New Genome Assembly Algorithm and Its Applications to Single-Cell Sequencing; Journal of Computational Biology. 2012; doi:10.1089/cmb.2012.0021

29. Simão FA, Waterhouse RM, loannidis P et al. BUSCO: Assessing Genome Assembly and Annotation Completeness with Single-Copy Orthologs. Bioinformatics. 2015; doi:10.1093/bioinformatics/btv351.

30. Finn RD, Clements J, Eddy SR et al. HMMER Web Server: Interactive Sequence Similarity Searching. Nucleic Acids Research. 2011; doi:10.1093/nar/gkr367.

31. Bushnell B. BBMap Short Read Aligner. Berkeley, CA: University of California; 2016. https://sourceforge.net/projects/bbmap/ (August 7 2018, date last accessed).

32. Walker BJ, Abeel T, Shea T et al. Pilon: an integrated tool for comprehensive microbial variant detection and genome assembly improvement. PLoS One. 2014; doi:10.1371/journal.pone.0112963

33. Langmead B, Salzberg SL. Fast Gapped-Read Alignment with Bowtie 2. Nature Methods. 2012; doi:10.1038/nmeth.1923.

34. Francis WR *haplotypeblastn.py;* https://bitbucket.org/wrf/sequences/raw/f23b4dd3c965cc1774b9e10eb433242a18c13c65/haplotypeblastn.py (August 7 2018, date last accessed).

35. Hahn C *select_contigs.pl;* https://github.com/chrishah/phylog/blob/master/scripts-external/select_contigs.pl (August 7 2018, date last accessed).

36. Smit AFA, Hubley R, Green P. RepeatMasker; http://repeatmasker.org

37. Lunter G, Goodson M. Stampy: a statistical algorithm for sensitive and fast mapping of Illumina sequence reads. Genome Research. 2011;21(6):936-939.

38. Pena-Centeno T; *filterBam,* https://github.com/nextgenusfs/augustus/tree/master/auxprogs/filterBam

39. https://computationalbiologysite.wordpress.com/2013/07/25/incorporating-rnaseq-tophat-to-augustus, (August 7 2018, date last accessed).

40. Stanke M, Steinkamp R, Waack S et al. AUGUSTUS: a web server for gene finding in eukaryotes. Nucleic Acids Research 2004;32(suppl–2):W309–12.

41. Stanke M, Schöffmann O, Morgenstern B□ Waack S. Gene prediction in eukaryotes with a generalized hidden Markov model that uses hints from external sources. BMC Bioinformatics. 2006; doi:10.1186/1471-2105-7-62.

42. Francis WR, Wörheide G. Similar ratios of introns to intergenic sequence across animal genomes. Genome Biology and Evolution; 2017;9(6):1582-1598.

43. Joint Genomics Institute: Trivial HTTP, Nemve1. https://genome.jgi.doe.gov/portal/Nemve1/Nemve1.download.ftp.html (7 August 2018, date last accessed)

44. Macdonald B. filterGenes.py. https://github.com/mcfaddenlab/filterGenes.py/blob/master/README.md (August 7, 2018, date last accessed)

45. Uniprot Consortium. UniProt: the Universal Protein Knowledgebase. Nucleic Acids Research. 2018; doi:10.1093/nar/gky092

46. UniProt Consortium, Reviewed Swiss-Prot data, ftp://ftp.uniprot.org/pub/databp/uniprot/current_release/knowledgebase/complete/uniprot_sprot.fasta.gz (August 7, 2018, date last accessed)

47. https://ndownloader.figshare.com/files/1215191, (August 7 2018, date last accessed).

48. Moran Y, Fredman D, Praher D et al. Cnidarian MicroRNAs frequently regulate targets by cleavage. Genome Research. 2014; doi:10.1101/gr.162503.113.

49. Joint Genome Institute. *Nematostella vectensis* genome. Version 1. https://genome.jgi.doe.gov/portal/Nemve1/Nemve1.download.html (August 7, 2018, date last accessed).

50. Putnam NH, Srivastava M, Hellsten U, Dirks B, Chapman J, Salamov, A, et al. Sea anemone genome reveals ancestral eumetazoan gene repertoire and genomic organization. Science 2007;317(5834):86-94.

51. Shinzato C, Shoguchi E, Kawashima T et al. National Center for Biotechnology Information, *Acropora digitifera* genome Version 1. https://www.ncbi.nlm.nih.gov/nuccore/BACK00000000.1 (November 2015, date last accessed).

52. Shinzato C, Shoguchi E, Kawashima T, et al. Using the *Acropora digitifera* genome to understand coral responses to environmental change. Nature. 2011;476:7360-320.

53. Liew YJ, Aranda M, Voolstra CR. Reefgenomics.Org - a repository for marine genomics data. Database (Oxford) 2016, 1–4 A*mplexidiscus fenestrafer* and *Discosoma* sp. genomes. http://corallimorpharia.reefgenomics.org (August 7, 2018, date last accessed).

54. Wang X, Liew YJ, Li Y, Zoccola D, Tambutte S, Aranda M. Draft genomes of the corallimorpharians *Amplexidiscus fenestrafer* and *Discosoma* sp. Molecular Ecology Resources 2017; 17(6); 187-195.

55. Baumgarten E, Simakov O, Esherick LY et al. National Center for Biotechnology Information, (*Ex*)*aiptasia pallida* genome Version 1.1 ftp://ftp.ncbi.nlm.nih.gov/sra/wgs_aux/LJ/WW/LJWW01/LJWW01.1.fsa_nt.gz (August 7 2018, date last accessed).

56. Baumgarten S, Simakov O, Esherick LY et al. The genome of *Aiptasia*, a sea anemone model for coral symbiosis. Proceedings of the National Academy of Sciences 2015;112(38):11893-11898.

57. Matz Lab. *Montastraea cavernosa* genome. Jul 2018 version. https://matzlab.weebly.com/data--code.html (August 7, 2018, date last accessed).

58. Kayal E, Bentlage B, Pankey MS et al. Phylogenomics provides a robust topology of the major cnidarian lineages and insights on the origins of key organismal traits. BMC Evolutionary Biology 2018;18:68.

59. Kayal E, Roure B, Phillipe H et al. Cnidarian phylogenetic relationships as revealed by mitogenomics. BMC Evolutionary Biology, 2013;13:5.

60. Jiang J, Quattrini AM, Francis WR, et al. A hybrid de novo assembly of the sea pansy (*Renilla muelleri)* genome. GigaScience Database 2018. doi:XXXXX

61. Liew YJ, Aranda M, Voolstra CR. Reefgenomics.Org - a repository for marine genomics data. Database (Oxford) 2016, 1–4 *Renilla muelleri* genome http://rmue.reefgenomics.org (August 7, 2018, date last accessed)

Figure Captions

Figure 1. BUSCO-generated chart showing relative completeness of six hexacoral genomes, one octocoral genome, and the *Renilla muelleri* assembly.

Table 1. General statistics and BUSCO-completeness of both initial hybrid assemblies and the final hybrid assembly.

	MaSuRCA hybrid	SPAdes hybrid	Final MaSuRCA hybrid
scaffold total	4,984	725,809	4,925
contig total	5,263	725,809	5,196
scaffold sequence total	172,512,580	231,255,108	172,160,214
contig sequence total	172.472 Mb	231.255 Mb	172.091 Mb
scaffold L/N50	635/70.423 Kb	33702/1.007 Kb	633/70.522 Kb
contig L/N50	687/64.492 Kb	33702/1.007 Kb	684/64.781 Kb
Max scaffold/contig length	513.145 Kb	323.009 Kb	513.151 Kb
Number of scaffolds > 50 Kb	960	14	961
% main genome in scaffolds > 50 Kb	61.07%	0.95%	61.23%
GC%	36.18%	36.97%	36.17%
N%	0.042%	0.000%	0.040%
BUSCO assessment:			
Complete	858 (87.73%)	508 (51.94%)	857 (87.63%)
Complete and single-copy	826 (84.46%)	493 (50.41%)	826 (84.46%)
Complete and Duplicated	32 (3.27%)	15 (1.53%)	31 (3.17%)
Fragmented	36 (3.68%)	200 (20.45%)	36 (3.68%)
Missing	84 (8.59%)	270 (27.61%)	85 (8.69%)

Unmerged haplotypes were removed in the final assembly, which was also error-corrected with Pilon.

Table 2. Statistics for the gene model predicted by Augustus**.**

	Number
Genes	23,660
Exons	140,384
Introns	117,838
Average Exon Length	249
Exons Per Gene	5.93
Average Intron Length	524
Introns Per Gene	4.98
BUSCO assessment:	
Complete	830 (84.87%)
Complete and single-copy	798 (81.60%)
Complete and Duplicated	32 (3.27%)
Fragmented	64 (6.54%)
Missing	84 (8.59%)

Supplemental Table S1. Summary of Pilon changes per iteration

	First Iteration	Second Iteration	Third Iteration	Fourth Iteration	Fifth Iteration	Sixth Iteration
Single-nucleotide polymorphism changes	32,292	10,039	4,688	2,790	1,697	1,152
Ambiguous bp	567	199	99	50	41	26
Small Insertions	9,180 (54,855 bp)	1,982 (15,381 bp)	1,231 (14,443 bp)	858 (11,391 bp)	810 (12,777 bp)	641 (10,596 bp)
Small Deletions	6706 (41,808 bp)	1,925 (16,566 bp)	1038 (11,922 bp)	848 (12,916 bp)	640 (10,603 bp)	684 (12,319 bp)

Supplemental Table S2. *Renilla muelleri* genome assembly and annotation comparisons to other anthozoan genomes.

	Genome Size (Mb)	Total # Complete BUSCOs**	Contig N50 (KB)	Exon length (bp)	# Predicted Gene models
Acropora digitifera	420	728	10.6	230	23,668
Amplexidiscus fenestrafer	350	816	20.0	218	21,372
Discosoma sp.	428	839	18.7	226	23,199
Exaiptasia pallida	256	833	14.4	NA	26,042
Montastraea cavernosa	448	800	343	NA	30,360
*Nematostella vectensis**	329	893	19.8	208	27,273
Renilla reniformis	132	356	1.8	NA	12,689
Renilla muelleri	172	857	64.8	249	23,360

* Data taken from [52] and [56]
**Complete BUSCOs generated from analysis herein

 Click here to download Figure Jiang_Figure1.png

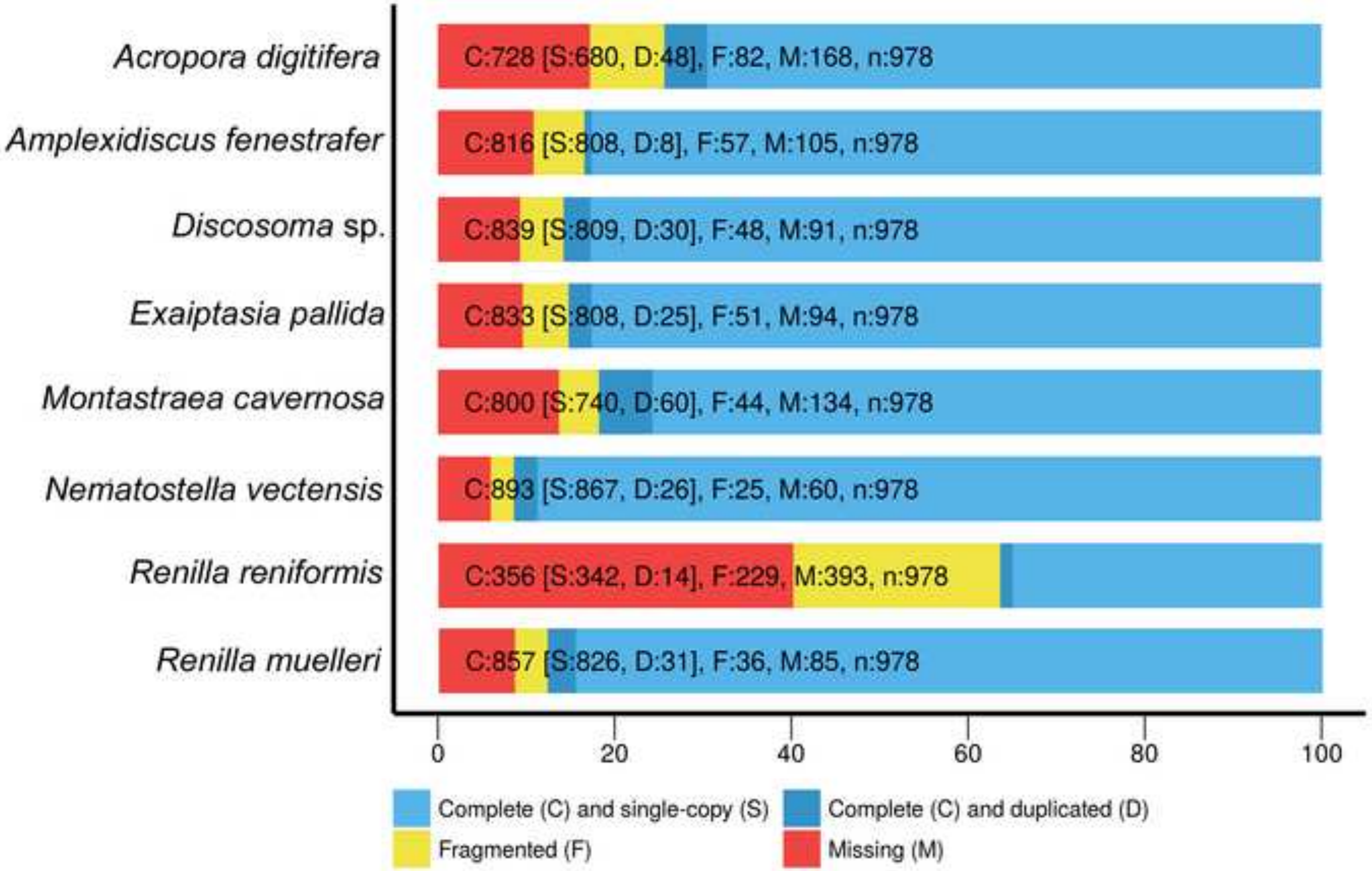

Click here to access/download
Supplementary Material
Jiang_suppltable_s1.docx

Click here to access/download
Supplementary Material
Jiang_suppltable_s2.docx

Click here to access/download
Supplementary Material
Jiang_SupplFile3.xlsx

Click here to access/download
Supplementary Material
Jiang_SupplFile1.xlsx

Click here to access/download
Supplementary Material
Jiang_SupplFile2.xlsx

Personal Cover Click here to download Personal Cover coverletter_gigasci.docx

20 Sep 2018

Dear Editor,

Included please find the original manuscript “A Hybrid *de novo* Assembly of the Sea Pansy (*Renilla muelleri*) Genome.” My co-authors and I present a novel genomic resource to advance the state of anthozoan (coral, anemone) genomics. Herein, we present a *de novo* assembly of an octocoral using Pacific Biosciences and Illumina data. We present one of the smallest coral genomes assembled to date and the only complete octocoral genome.

We feel that this manuscript is a prefect fit as a data note in *GigaScience* and that it will have broad appeal to the large audience of this journal. On the next page, we list suggested reviewers that have been involved in genome assemblies of other anthozoans.

Please consider this manuscript for review.

Sincerely,

Andrea M. Quattrini
aquattrini@g.hmc.edu
910-520-7264

Suggested Reviewers

1. Yi Jen Liew, KAUST, yijin.liew@kaust.edu.sa
2. Sebastian Baumgarten, Institut Pasteur, sebastian.baumgarten@pasteur.fr
3. Chuya Shinzato, University of Tokyo, c.shinzato@aori.u-tokyo.ac.jp
4. Mikhail Matz, University Texas Austin, matz@utexas.ed
