# Supplementary material for: A hybrid de novo assembly of the sea pansy (Renilla muelleri) genome
Source: Gigascience. 2019 Apr 3;8(4):giz026. doi: 10.1093/gigascience/giz026 (PMC6446218; doi:10.1093/gigascience/giz026)
Supplement: Supplement_Files.zip [file giz026_supplement_files.zip › Jiang_suppltable_s1.docx]

**Supplemental Table S1.** Summary of Pilon changes per iteration

|  | First Iteration | Second Iteration | Third Iteration | Fourth Iteration | Fifth Iteration | Sixth Iteration |
| --- | --- | --- | --- | --- | --- | --- |
| Single-nucleotide polymorphism changes | 32,292 | 10,039 | 4,688 | 2,790 | 1,697 | 1,152 |
| Ambiguous bp | 567 | 199 | 99 | 50 | 41 | 26 |
| Small Insertions | 9,180  (54,855 bp) | 1,982 (15,381 bp) | 1,231  (14,443 bp) | 858  (11,391 bp) | 810 (12,777 bp) | 641 (10,596 bp) |
| Small Deletions | 6706  (41,808 bp) | 1,925 (16,566 bp) | 1038  (11,922 bp) | 848  (12,916 bp) | 640 (10,603 bp) | 684 (12,319 bp) |
