# Supplementary material for: A hybrid de novo assembly of the sea pansy (Renilla muelleri) genome
Source: Gigascience. 2019 Apr 3;8(4):giz026. doi: 10.1093/gigascience/giz026 (PMC6446218; doi:10.1093/gigascience/giz026)
Supplement: Supplement_Files.zip [file giz026_supplement_files.zip › Jiang_suppltable_s2.docx]

**Supplemental Table S2.** *Renilla muelleri* genome assembly and annotation comparisons to other anthozoan genomes.

|  | Genome Size (Mb) | Total # Complete BUSCOs** | Contig N50 (KB) | Exon length (bp) | # Predicted Gene models |
| --- | --- | --- | --- | --- | --- |
| *Acropora digitifera* | 420 | 728 | 10.6 | 230 | 23,668 |
| *Amplexidiscus fenestrafer* | 350 | 816 | 20.0 | 218 | 21,372 |
| *Discosoma* sp. | 428 | 839 | 18.7 | 226 | 23,199 |
| *Exaiptasia pallida* | 256 | 833 | 14.4 | NA | 26,042 |
| *Montastraea cavernosa* | 448 | 800 | 343 | NA | 30,360 |
| *Nematostella vectensis** | 329 | 893 | 19.8 | 208 | 27,273 |
| *Renilla reniformis* | 132 | 356 | 1.8 | NA | 12,689 |
| *Renilla muelleri* | 172 | 857 | 64.8 | 249 | 23,360 |

* Data taken from [52] and [56]

**Complete BUSCOs generated from analysis herein
