# Supplementary material for: A hybrid de novo assembly of the sea pansy (Renilla muelleri) genome
Source: Gigascience. 2019 Apr 3;8(4):giz026. doi: 10.1093/gigascience/giz026 (PMC6446218; doi:10.1093/gigascience/giz026)
Supplement: Response-to-Reviewer-Comments_Original-Submission.pdf [file giz026_response-to-reviewer-comments_original-submission.pdf]

Author's Response To Reviewer Comments

Close

15 Jan 2019
Dear Editor,

We thank you and the two reviewers for edits and suggestions that improved our paper, “A Hybrid de novo Assembly of the Sea Pansy (Renilla muelleri) Genome”. We took all suggestions; below you will find our replies to individual comments. We are confident that the additional changes have strengthened the overall quality of our work.

I hope you find the changes acceptable for publication.

Cheers,

Andrea Quattrini
Corresponding author

GIGA-D-18-00366
A Hybrid de novo Assembly of the Sea Pansy (Renilla muelleri) Genome
Justin Jiang; Andrea M. Quattrini; Warren R. Francis; Joseph F. Ryan; Estefanía Rodríguez; Catherine S. McFadden
GigaScience

From the Editor:

The assembly is rather fragmented, compared to the standard of many of our other data notes published in the journal, but considering that genomic information on corals is sparse, we agree with the reviewers that the work has merit as a data note, in principle.
>Thank you for seeing the value in this work!

However, if you can further improve the work prior to publication this would be a plus. In particular, please address the issues of genome size estimation (which may need more careful wording) and potential contamination - see the reviewers' reports.
>Please see answers below.

Reviewer reports:
Reviewer #1: This manuscript describes the production of a genome assembly for the octocoral Renilla muelleri. The assembly makes use of a combination of Illumina and PacBio reads to achieve the final assembly. The value of this manuscript is in the fact that it presents the genome from an octocoral, which have not been well sampled by genome sequencing despite their ecological importance. Specific points that need to be addressed are as follows:

1. The authors state that the assembly and predicted proteins are in GigaDB. I could not find an entry for these data in GigaDB.
>Editor stated that this was a confusion and our data will be included.

2. The authors state that their assembly is the "first complete draft genome from an octocoral." But they also state that a genome for Renilla reniformis has already been published. So how can their genome assembly be first, if there is already a published genome from Renilla?
>We reworded this as to not include “first”, instead this reads “Here, we present a de novo assembly of an azooxanthellate shallow-water octocoral, R. muelleri.” It is true that the genome of Renilla reniformis exists, but it is even more fragmented than ours and contains few complete BUSCOS.

3. To identify and remove reads from organisms contaminating their Renilla sample, the authors used screening against the NCBI environmental nucleotide database. How effective is this? It seems to me that this would not be a very effective way to remove contaminating reads since it will only identify reads that are relatively similar at the nucleotide sequence level to those in the database. For example if their Renilla sample contains bacteria that are not closely related to those whose sequences are in the environmental nucleotide database, will these be removed? Can the authors provide some bioinformatic data that show
that bacterial sequences have been effectively removed from the assembly?
>The reviewer raises a very good point in that contamination removal is only as good as the database. However, to our knowledge, few studies have attempted to eliminate microbial contaminants from invertebrate genomes, which all house extensive microbial communities, prior to assembly. Recently, Voolstra et al. 2017 removed scaffolds (so after assembly) that blasted to environmental contaminants at an e value of e-20. Thus, our cutoff was more stringent, and removal occurred prior to assembly. We feel that we did an adequate job at removing contaminants from the illumina data using Kraken (which uses RefSeq microbial genomes), which does not match up nucleotides but rather takes a kmer alignment approach, and from the PacBio data using a screening against env_nt database. We are unsure of what types of bioinformatic data could show how effective contaminant removal was, as we do not know the entire microbial consortia living on Renilla; however, we did include all of the read names that were removed in the supplemental material, and an xml file of the pacbio read blast results.

4. On line 169 of page 8, the authors used the term "intron hints." What does this mean?
>We added, "which provide evidence for introns based on spliced alignments"

5. On page 10, the authors describe the Nematostella genome assembly as "well-curated." In fact, the public Nematostella genome assembly is still at version 1.0, and it has only undergone one pass of automated annotation. I would not consider it well-curated.
>Valid point. We removed well-curated.

6. On line 232 on page 11, the authors state that the "genome size of R. muelleri is considerably small (172 Mb) than other hexacoral genomes." This wording implies that R. muelleri is a hexacoral.
>Corrected to coral genomes

7. If I am reading Table 1 correctly, the scaffold N50 (70.5 kb) is only slightly larger than the contig N50 (64.8 kb). Why so little improvement between contigs and scaffolds?
>More data are necessary to improve this We made that clear on lines 257-258 "Although more data are needed to further increase size and reduce number of scaffolds"

Reviewer #2: This is a basic description of the first genome draft assembly of the R. muelleri genome. The article describes the data collection, genome assembly and annotation of R. muelleri, but makes no attempt to extract any real biology, rather it is presented as a resource to the community. The article is well written and includes the GigaScience minimal reporting standards. The assembly strategy seems reasonable. The only concern I have is the statement that the R. muelleri genome is 172Mb (and indeed that the genomes of other hexcorals are 256-448Mb). This needs to be changed to state that the R. muelleri genome is at least 172Mb. The genome draft consists of 4,925 scaffolds, which is obviously much higher than the number of chromosomes of this species. Although I do not think we have much knowledge on the chromosome complements of corals (at least I found very little when I tried various searches), there is clearly more work to do to contiguate all the scaffolds
into chromosomes. This will no doubt will increase the genome size. The same is true of the other corals, which are even more fragmented than that of R. muelleri and therefore their genomes sizes are also minimum estimates.
>We added "Although the R. muelleri genome may be smaller (172 Mb minimum size) than other publicly available, coral genomes (256-448 Mb)" to the abstract, lines 40-41 and "although these genome sizes are minimum estimates due to the high number of scaffolds and fragmentary nature of the assemblies" To lines 235-236.

Close
