# Supplementary material for: A hybrid de novo assembly of the sea pansy (Renilla muelleri) genome
Source: Gigascience. 2019 Apr 3;8(4):giz026. doi: 10.1093/gigascience/giz026 (PMC6446218; doi:10.1093/gigascience/giz026)
Supplement: Reviewer-1-Report-Original-Submission -- Robert Steele, Ph.D. — 12/5/2018 Reviewed [file giz026_reviewer-1-report-original-submission.pdf]

Reviewer Report

Title: A Hybrid de novo Assembly of the Sea Pansy (Renilla muelleri) Genome

Version: Original Submission **Date:** 12/5/2018

Reviewer name: Robert Steele, Ph.D.

Reviewer Comments to Author:

This manuscript describes the production of a genome assembly for the octocoral Renilla muelleri. The assembly makes use of a combination of Illumina and PacBio reads to achieve the final assembly. The value of this manuscript is in the fact that it presents the genome from an octocoral, which have not been well sampled by genome sequencing despite their ecological importance. Specific points that need to be addressed are as follows:

1. The authors state that the assembly and predicted proteins are in GigaDB. I could not find an entry for these data in GigaDB.
2. The authors state that their assembly is the "first complete draft genome from an octocoral." But they also state that a genome for Renilla reniformis has already been published. So how can their genome assembly be first, if there is already a published genome from Renilla?
3. To identify and remove reads from organisms contaminating their Renilla sample, the authors used screening against the NCBI environmental nucleotide database. How effective is this? It seems to me that this would not be a very effective way to remove contaminating reads since it will only identify reads that are relatively similar at the nucleotide sequence level to those in the database. For example if their Renilla sample contains bacteria that are not closely related to those whose sequences are in the environmental nucleotide database, will these be removed? Can the authors provide some bioinformatic data that show that bacterial sequences have been effectively removed from the assembly?
4. On line 169 of page 8, the authors used the term "intron hints." What does this mean?
5. On page 10, the authors describe the Nematostella genome assembly as "well-curated." In fact, the public Nematostella genome assembly is still at version 1.0, and it has only undergone one pass of automated annotation. I would not consider it well-curated.
6. On line 232 on page 11, the authors state that the "genome size of R. muelleri is considerably small (172 Mb) than other hexacoral genomes." This wording implies that R. muelleri is a hexacoral.
7. If I am reading Table 1 correctly, the scaffold N50 (70.5 kb) is only slightly larger than the contig N50 (64.8 kb). Why so little improvement between contigs and scaffolds?

Level of Interest

Please indicate how interesting you found the manuscript: Choose an item.

Quality of Written English

Please indicate the quality of language in the manuscript: Choose an item.

Declaration of Competing Interests

Please complete a declaration of competing interests, considering the following questions:

- Have you in the past five years received reimbursements, fees, funding, or salary from an organisation that may in any way gain or lose financially from the publication of this manuscript, either now or in the future?
- Do you hold any stocks or shares in an organisation that may in any way gain or lose financially from the publication of this manuscript, either now or in the future?
- Do you hold or are you currently applying for any patents relating to the content of the manuscript?
- Have you received reimbursements, fees, funding, or salary from an organization that holds or has applied for patents relating to the content of the manuscript?
- Do you have any other financial competing interests?
- Do you have any non-financial competing interests in relation to this paper?

If you can answer no to all of the above, write 'I declare that I have no competing interests' below. If your reply is yes to any, please give details below.

I declare that I have no competing insterests

I agree to the open peer review policy of the journal. I understand that my name will be included on my report to the authors and, if the manuscript is accepted for publication, my named report including any attachments I upload will be posted on the website along with the authors' responses. I agree for my report to be made available under an Open Access Creative Commons CC-BY license (http://creativecommons.org/licenses/by/4.0/). I understand that any comments which I do not wish to be included in my named report can be included as confidential comments to the editors, which will not be published.

I agree to the open peer review policy of the journal

To further support our reviewers, we have joined with Publons, where you can gain additional credit to further highlight your hard work (see: https://publons.com/journal/530/gigascience). On publication of this paper, your review will be automatically added to Publons, you can then choose whether or not to claim your Publons credit. I understand this statement.

Yes Choose an item.
