# Supplementary material for: A hybrid de novo assembly of the sea pansy (Renilla muelleri) genome
Source: Gigascience. 2019 Apr 3;8(4):giz026. doi: 10.1093/gigascience/giz026 (PMC6446218; doi:10.1093/gigascience/giz026)
Supplement: Reviewer-2-Report-Original-Submission -- Melody Clark — 12/13/2018 Reviewed [file giz026_reviewer-2-report-original-submission.pdf]

Reviewer Report

Title: A Hybrid de novo Assembly of the Sea Pansy (Renilla muelleri) Genome

Version: Original Submission **Date:** 12/13/2018

Reviewer name: Melody Clark

Reviewer Comments to Author:

This is a basic description of the first genome draft assembly of the R. muelleri genome. The article describes the data collection, genome assembly and annotation of R. muelleri, but makes no attempt to extract any real biology, rather it is presented as a resource to the community. The article is well written and includes the GigaScience minimal reporting standards. The assembly strategy seems reasonable. The only concern I have is the statement that the R. muelleri genome is 172Mb (and indeed that the genomes of other hexcorals are 256-448Mb). This needs to be changed to state that the R. muelleri genome is at least 172Mb. The genome draft consists of 4,925 scaffolds, which is obviously much higher than the number of chromosomes of this species. Although I do not think we have much knowledge on the chromosome complements of corals (at least I found very little when I tried various searches), there is clearly more work to do to contiguate all the scaffolds into chromosomes. This will no doubt will increase the genome size. The same is true of the other corals, which are even more fragmented than that of R. muelleri and therefore their genomes sizes are also minimum estimates.

Level of Interest

Please indicate how interesting you found the manuscript: Choose an item.

Quality of Written English

Please indicate the quality of language in the manuscript: Choose an item.

Declaration of Competing Interests

Please complete a declaration of competing interests, considering the following questions:

- Have you in the past five years received reimbursements, fees, funding, or salary from an organisation that may in any way gain or lose financially from the publication of this manuscript, either now or in the future?
- Do you hold any stocks or shares in an organisation that may in any way gain or lose financially from the publication of this manuscript, either now or in the future?
- Do you hold or are you currently applying for any patents relating to the content of the manuscript?
- Have you received reimbursements, fees, funding, or salary from an organization that holds or has applied for patents relating to the content of the manuscript?

- Do you have any other financial competing interests?
- Do you have any non-financial competing interests in relation to this paper?

If you can answer no to all of the above, write 'I declare that I have no competing interests' below. If your reply is yes to any, please give details below.

I declare that I have no competing interests

I agree to the open peer review policy of the journal. I understand that my name will be included on my report to the authors and, if the manuscript is accepted for publication, my named report including any attachments I upload will be posted on the website along with the authors' responses. I agree for my report to be made available under an Open Access Creative Commons CC-BY license (http://creativecommons.org/licenses/by/4.0/). I understand that any comments which I do not wish to be included in my named report can be included as confidential comments to the editors, which will not be published.

I agree to the open peer review policy of the journal

To further support our reviewers, we have joined with Publons, where you can gain additional credit to further highlight your hard work (see: https://publons.com/journal/530/gigascience). On publication of this paper, your review will be automatically added to Publons, you can then choose whether or not to claim your Publons credit. I understand this statement.

Yes Choose an item.
